# Receptor Activator of NF-kappaB and Podocytes: Towards a Function of a Novel Receptor-Ligand Pair in the Survival Response of Podocyte Injury

**DOI:** 10.1371/journal.pone.0041331

**Published:** 2012-07-25

**Authors:** Shuangxin Liu, Wei Shi, Houqin Xiao, Xinling Liang, Chunyu Deng, Zhiming Ye, Ping Mei, Suxia Wang, Xiaoying Liu, Zhixin Shan, Yongzheng Liang, Bin Zhang, Wenjian Wang, Yanhui Liu, Lixia Xu, Yunfeng Xia, Jianchao Ma, Zhilian Li

**Affiliations:** 1 Department of Nephrology, Guangdong General Hospital, Guangdong Academy of Medical Sciences, Guangzhou, Guangdong, China; 2 Southern Medical University, Guangzhou, Guangdong, China; 3 Medical Research Center, Guangdong General Hospital, Guangdong Academy of Medical Sciences, Guangzhou, Guangdong, China; 4 Department of Pathology, Guangdong General Hospital, Guangdong Academy of Medical Sciences, Guangzhou, Guangdong, China; 5 Department of Pathology, Peking University First Hospital, Beijing, China; University of Pecs Medical School, Hungary

## Abstract

**Background:**

Glomerulosclerosis correlates with reduction in podocyte number that occurs through mechanisms which include apoptosis. Podocyte injury or podocyte loss in the renal glomerulus has been proposed as the crucial mechanism in the development of glomerulosclerosis. However, the mechanism by which podocytes respond to injury is poorly understood. TNF and TNF receptor superfamilies are important in the pathogenesis of podocyte injury and apoptosis. The ligand of receptor activator of NF-kappaB (RANKL) and receptor activator of NF-kappaB (RANK) are members of the TNF and receptor superfamilies. We investigated whether RANK - RANKL is a receptor - ligand complex for podocytes responding to injury.

**Methodology/Principal Findings:**

In this study, RANKL and RANK were examined in human podocyte diseases and a rat model of puromycin aminonucleoside nephrosis (PAN). Compared with controls, RANK and RANKL were increased in both human podocyte diseases and the rat PAN model; double immunofluorescence staining revealed that RANK protein expression was mainly attributed to podocytes. Immunoelectron microscopy showed that RANK was localized predominantly at the top of the foot process membrane and the cytoplasm of rat podocyte. In addition, RANK was upregulated in mouse podocytes *in vitro* after injury induced by puromycin aminonucleoside (PA). Knockdown of RANK expression by small interference RNA (siRNA) exacerbated podocyte apoptosis induced by PA. However, RANKL inhibited significantly the apoptosis of podocytes induced by PA.

**Conclusions/Significance:**

These findings suggest the increase in RANK–RANKL expression is a response to podocyte injury, and RANK–RANKL may be a novel receptor–ligand complex for the survival response during podocyte injury.

## Introduction

Podocytes are terminally differentiated cells that line the outer aspect of the glomerular basement membrane (GBM) [1]. Podocyte dysfunction, injury, or loss is a common and determining factor in glomerular diseases [2]. Extensive experimental and clinical data have confirmed the importance of podocyte injury in the development and progression of glomerular disease [3]–[4]. Podocytes are believed to be the primary target of glomerular damage in so-called podocytopathies (minimal change disease (MCD) [5], focal segmental glomerulosclerosis (FSGS) [6], and membranous nephropathy (MN) [7]), as well they are also damaged in glomerular diseases of mesangial proliferation, including IgA nephropathy and lupus nephritis [8]–[10].

Podocytes are injured in immune- and non-immune-mediated disease, resulting in damage to the glomerular filtration barrier. The fate of the podocyte then depends on several factors, such as reparative mechanisms and injury factors. If these are present, and/or the initial injury is halted, there may be resolution. However, if injury persists, and/or there are inadequate repair mechanisms present, proteinuria persists, leading to reduced renal function [11]. During the injury process, there is a critical period of coordinated gene expression that determines whether podocytes survive or lose [12]. In response to injury, podocytes secrete antioxidant enzymes [13] and abnormal proteins [14], such as desmin [15] and glial cell line-derived neurotrophic factor (GDNF) [16], which is a survival growth factor for injured podocytes. However, the pathogenesis of podocyte injury is not quite clear. We postulate that there are other survival factors that are expressed in response to podocyte injury and act to support the recovery of injured podocytes.

TNF and TNF receptor superfamilies are important in the pathogenesis of podocyte injury and apoptosis [17]–[18]. The ligand of receptor activator of NF-kappaB (RANKL) is a member of the TNF family [19] that is produced by osteoblasts, myocardial [20] and stromal cells [21]. RANKL is not only a transmembrane molecule but also secreted particularly by activated T cells [22]. Receptor activator of NF-kappaB (RANK) is a cognate receptor which is expressed by osteoclast-like cells (OCLS). We don’t know whether RANK–RANKL is a receptor–ligand complex for pathogenesis of podocyte injury and apoptosis. Several studies suggest that RANKL and RANK are involved in cell survival and apoptosis [23]. RANK expression has been shown to suppress endothelial cell apoptosis through its activation by RANKL [24]. RANK-RANKL is expressed not only in bone marrow-derived cells but also in non-bone marrow-derived cells. In human myocardial cells, RANK–RANKL gene expression is upregulated by allergens and irritants [20]. RANK is expressed in skin and mammary epithelial cells. RANKL is also expressed in lymph node stromal cell [25], skin epithelial cell [26], renal glomeruli, convoluted tubules, and parenchyma of the developing fetal kidney, whereas RANKL is not detected in adult kidney [27]. However, no studies have addressed the functional role of RANK–RANKL in normal renal physiology, or in glomerular disease. We report here for the first time that RANKL and RANK are induced significantly in animal models of podocyte injury. Moreover, RANKL, acting through RANK, is a potent survival factor for injured podocytes and promotes protection from injury.

## Results

### Identification of RANK and RANKL as Genes Upregulated in the Rats Podocyte Injury Model

**Figure 1:**
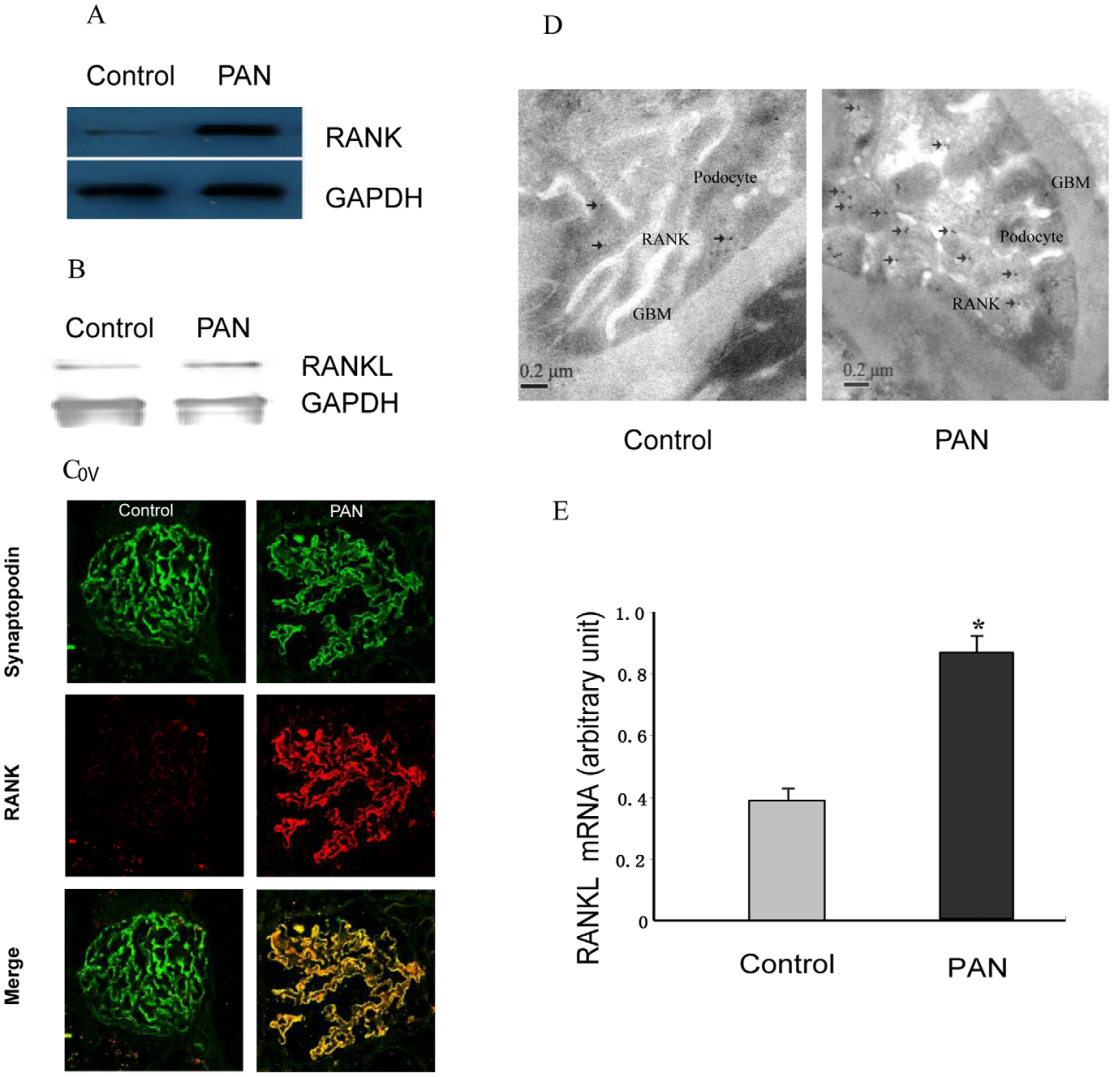
Identification of RANK and RANKL as Upregulation Genes in the Rat Podocyte Injury Model. (A) RANK protein expression was determined by using Western blot analysis with a RANK antibody. GAPDH immunoblotting served as a loading control and confirmed equal protein loading between samples. RANK protein was in low abundance in the rat control. By contrast, RANK increased in PAN models (5.8±0.7-fold of controls, n = 6, p<0.01). (B) The levels of RANKL protein increased in PAN rat kidney as compared with control. (C) Induction of RANK was revealed in podocytes in the PAN model using immunofluorescence. RANK expression (red) was low in control glomeruli from rat and colocalized with the podocyte marker synaptopodin (green). In podocytes from animals with PAN, RANK expression was substantially upregulated in podocytes, resulted in a yellow overlap with synaptopodin (Merge). (D) Immunogold analysis of RANK in glomerular walls of normal and PAN rats. RANK was substantially induced in the top of the foot process membrane and the cytoplasm of podocytes in PAN rats. Black arrows mark RANK in podocytes. (E) Induction of RANKL mRNA was upregulated in PAN rats kidney. Quantitative real-time RT-PCR was performed on kidney from PAN rats (2.6±0.4-fold of controls, n = 6). *Compared with control, p<0.01.

PAN is used widely as a model of podocyte injury [28]. To determine whether RANK and RANKL are increased in response to podocyte injury *in vivo*, we measured the expression of RANK and RANKL in the PAN rat model. PAN induced by a single injection of PA was characterized by an increase in urine protein levels at 7 days of follow-up (135±29 versus 6.15±0.68 mg/24 h; p<0.01). We found that rats with PAN had significantly increased levels of RANK protein in the kidney compared with controls (5.8±0.7-fold higher than controls; n = 6; p<0.01), with persistently raised levels throughout the observation period (Figure 1A). RANKL did not display a similar increase in protein levels as that observed for RANK (Figure 1B). RANK was localized in podocytes, as indicated by co-labeling with synaptopodin [29], a marker of podocyte (Figure 1C). Compared with controls, immunofluorescence staining for RANK was increased in PAN, and double immunofluorescence staining revealed that the increase in RANK protein expression was mainly attributed to its increase in podocyte. We also found low expression of RANK in proximal tubular cells appeared unchanged under normal and disease conditions (data not shown). To define the subcellular localization of RANK within podocytes, we performed immunogold analysis of RANK in glomerular walls of normal and PAN rats. Under normal conditions, low RANK expression was observed in podocytes, but increased RANK labeling was located on the top of the foot process membrane and the cytoplasm of podocytes in PAN rats (Figure 1D). Significantly increased RANKL mRNA levels were observed in PAN rats after 7 days (2.6±0.4-fold higher than controls, n = 6; p<0.01) (Figure 1E).

### RNA Expression of RANK and RANKL in Human Podocyte Injury Diseases

**Figure 2:**
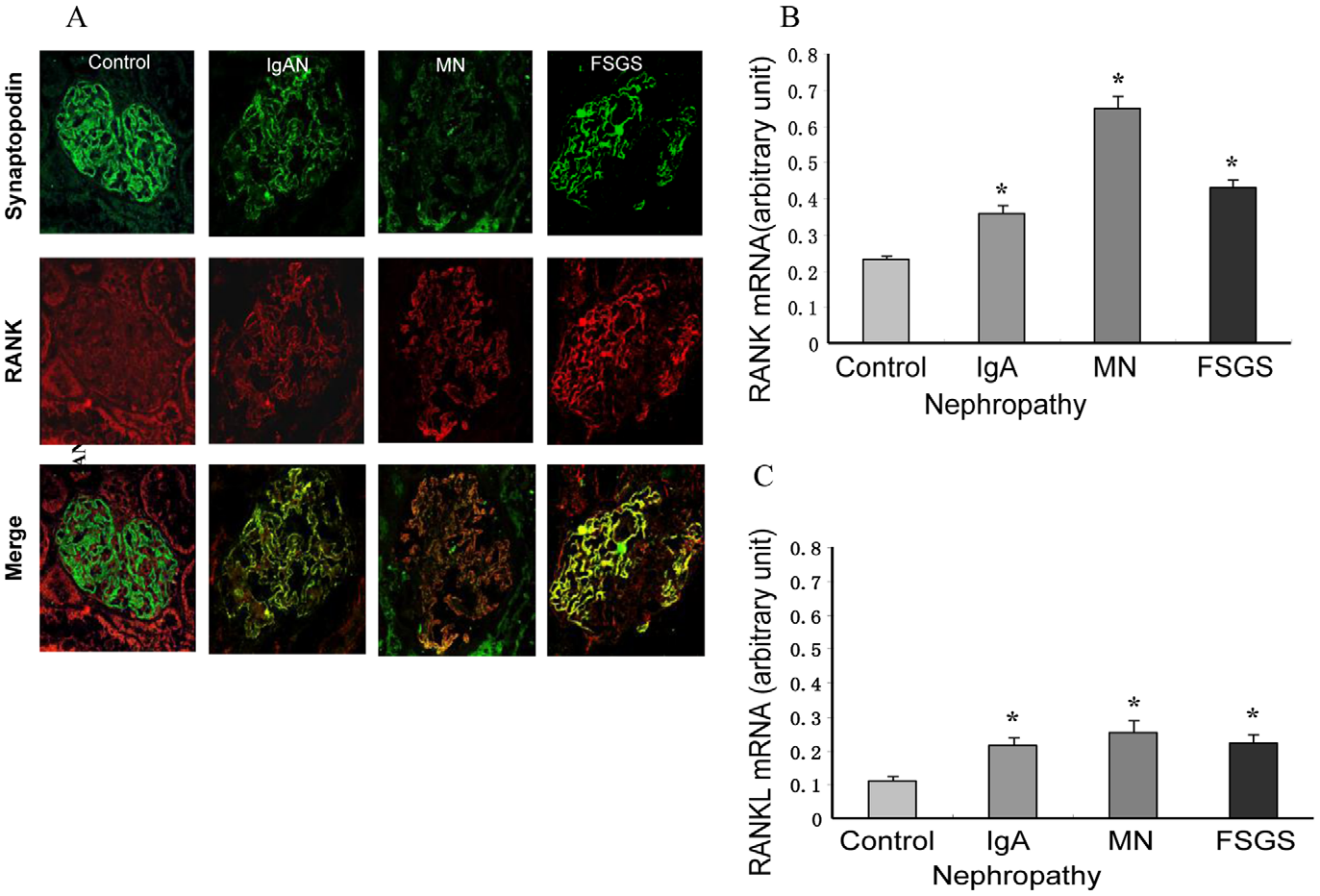
Expression of RANK and RANKL in Human Podocyte Injury Diseases. (A) RANK protein (red) was expressed in glomeruli of human kidney. RANK was found in podocytes, as shown by double immunofluorescence with the podocyte marker synaptopodin (green) resulting in yellow overlap (Merge). Compared with control, immunofluorescence staining for RANK was increased in IgA nephropathy, MN and FSGS, double immunofluorescence staining revealed that the increase in RANK protein expression was mainly attributed to podocytes. There was no immunfluoresence in the glomerulosclerosis position of FSGS. (B) RANK mRNA was expressed in human kidneys. Quantitative real-time RT-PCR was performed on kidney tissue from human biopsies. RANK mRNA was low in individuals without glomerular disease. By contrast, individuals with biopsy-proven FSGS, IgA nephropathy and MN had a significant increase in RANK expression (FSGS 0.42±0.07, IgA nephropathy 0.37±0.10, MN 0.67±0.24, versus control 0.24±0.04). *Compared with control, p<0.01. (C) Quantitative real-time RT-PCR was performed on kidney tissue from human biopsies. RANKL mRNA was low in individuals without glomerular disease. By contrast, individuals with FSGS, IgA nephropathy and MN had a moderate increase in RANKL expression (FSGS 0.25±0.07, IgA nephropathy 0.22±0.08, MN 0.27±0.08, versus control 0.11±0.05). *Compared with control, p<0.01.

Our findings suggest that the expression of RANK and RANKL are increased in an experimental rat model of podocyte injury. We then examined the possible relevance of these findings in human podocyte injury diseases. We performed quantitative RT-PCR with human kidney biopsies. We analyzed mRNA expression of RANK and RANKL in normal kidney tissues (n = 3), focal segmental glomerulosclerosis (FSGS, n = 12), IgA nephropathy (n = 14), and membranous nephropathy (MN, n = 16), and the latter three groups are with proteinuria. Confocal microscopy showed the co-localization of RANK and synaptopodin, indicating that podocytes contribute to glomerular RANK expression in podocyte injury diseases (Figure 2A). We found low levels of RANK mRNA expression in individuals without glomerular disease. By contrast, individuals with biopsy-proven FSGS, IgA nephropathy and MN had a significant increase in RANK expression (FSGS 0.42±0.07, IgA nephropathy 0.37±0.10, MN 0.67±0.24, versus control 0.24±0.04, p < 0.01) (Figure 2B). We also found low levels of RANKL mRNA expression in individuals without glomerular disease. By contrast, individuals with FSGS, IgA nephropathy and MN had a moderate increase in RANKL expression (FSGS 0.25±0.07, IgA nephropathy 0.22±0.08, MN 0.27±0.08, versus control 0.11±0.05, p < 0.01) (Figure 2C).

### LPS-induced RANK is Independent of T or B Cells in Severe Combined Immunodeficiency (SCID) Mice

**Figure 3:**
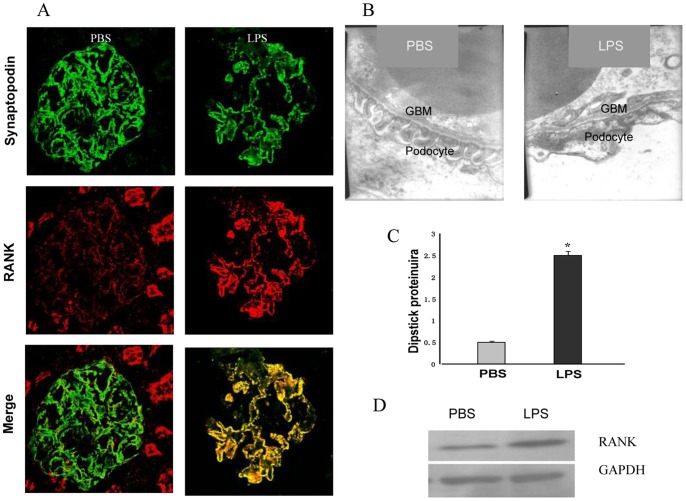
LPS-induced RANK was Independent of T or B Cell in SCID Mice. (**A**) LPS injection induces RANK in podocytes as shown by double labeling with synaptopodin. RANK expression (red) was low in PBS-injected SCID controls and colocalized with the podocyte marker synaptopodin (green). Mice treated with LPS, RANK expression was upregulated in podocytes, resulted in a yellow overlap with synaptopodin (Merge). (**B**) LPS injection causes severe foot process effacement within 24 h in mice, but not in PBS-injected control animals. (**C**) LPS injection causes severe proteinuria in SCID mice at 24 h (n = 3; PBS control 0.5±0.2 versus LPS 2.5±0.8; p<0.001). (**D**) RANK protein expression was determined by using Western blot analysis. RANK protein was in low abundance in the PBS group. However, RANK increased in the LPS group.

RANKL is a member of the tumor necrosis factor superfamily, which is expressed by activated T-cells. To determine whether RANK upregulation resulted from a direct action in podocytes rather than from the activation of T cells, we measured the expression of RANK in SCID mice treated with lipopolysaccharide (LPS), which are devoid of T and B cells [30]–[31]. LPS induced upregulation of RANK expression in podocytes of SCID mice (Figure 3A), and foot processes effacement (Figure 3B). Most significantly, LPS injection caused severe proteinuria at 24 hours (Figure 3C). Compared with PBS-injected SCID mice control, RANK was upregulated in LPS group (Figure 3D). Hence, induction RANK expression of podocytes in response to LPS was independent of T or B cells. Taken together, these data indicated that RANK expression in podocytes was independent of lymphocyte infiltration or activation.

### Expression of RANK in Cultured Differentiated Podocytes

**Figure 4:**
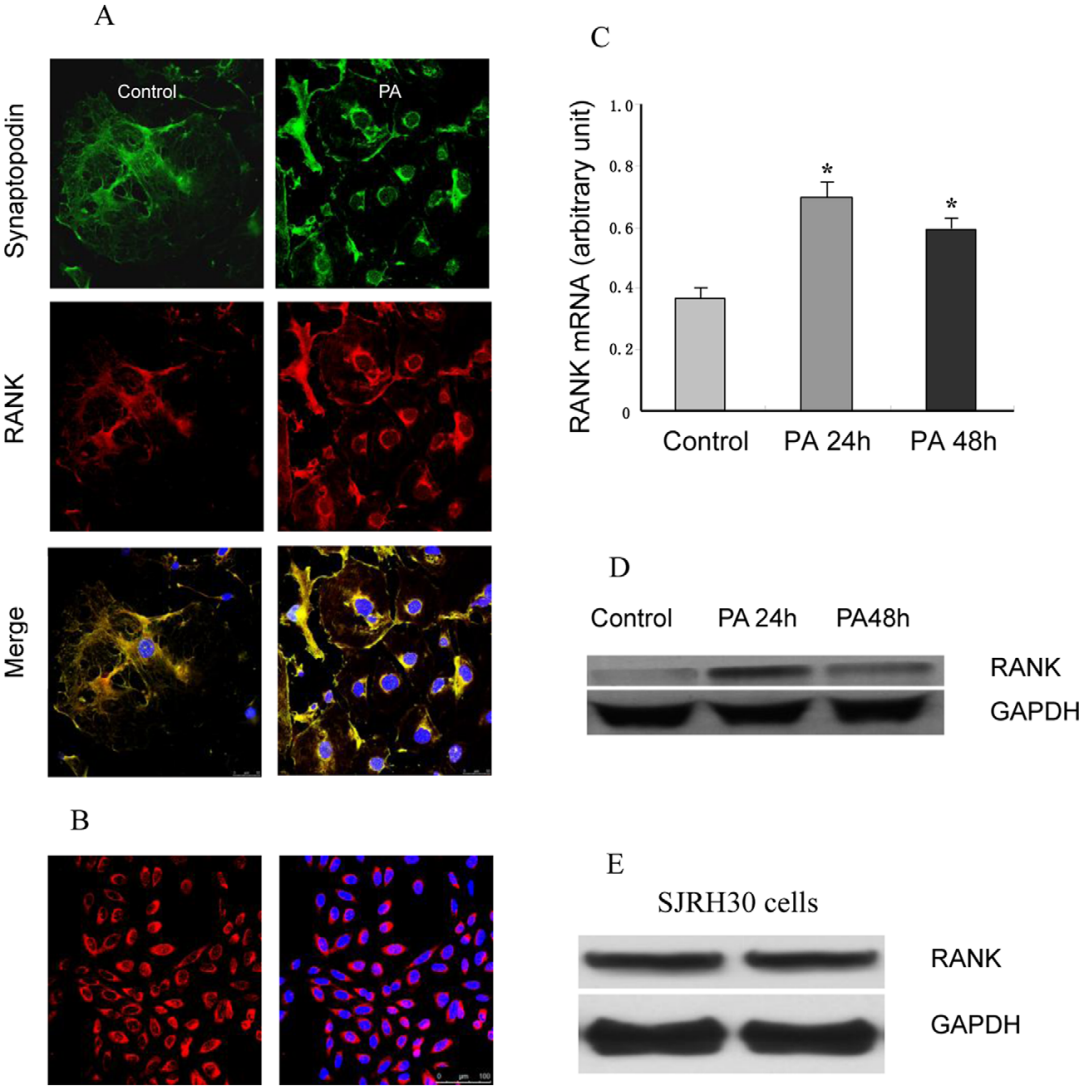
RANK Expression was Upregulated in a Model of Podocyte Injury in Vitro. (A) RANK expression co-localizes with synaptopodin. RANK expression was localized to the cell plasma membrane and cytoplasm. Synaptopodin (green) positive cells were positive for RANK (*red*) and showed morphology consistent with podocytes. Nuclei stained with DAPI (*blue*). (B) RANK positive controls are SJRH30 cells. RANK immunofluorescence staining of acetone-fixed SJRH30 cells showed membrane localization. Nuclei stained with DAPI (*blue*). (C) For determination of whether RANK was increased after PA (25 µg/ml) exposure, RANK mRNA was measured by real-time RT-PCR at 24 h and 48 h after PA exposure. Densitometric analyses indicate a 1.8- and 1.5-fold induction of RANK at 24 h and 48 h, respectively. Results were obtained from three independent experiments. *Compared with control, *P*<0.05. (D) Whole-cell lysates were harvested 24 or 48 h after PA injury or control conditions, and RANK protein expression was determined by using Western blot analysis with RANK antibody. Glyceraldehyde-3-phosphate dehydrogenase (GAPDH) immunoblotting served as a loading control and confirmed equal protein loading between samples. RANK protein was in low abundance in the absence of PA injury. In contrast, RANK expression was increased after PA injury at 24 h *and* 48 h. Densitometric analyses revealed that RANK was upregulated a 3.8- and 2.4-fold at 24 h and 48 h, respectively. RANK immunoblotting revealed that RANK is activated in an autocrine manner upon PA injury in podocytes, reaching a maximum within 24 h of PA treatment. (E) SJRH30 cells are RANK positive controls. RANK protein was confirmed by Western blot in SJRH30 cells.

Immortalized mouse podocytes were used and differentiated by raising the cell culture temperature from 33° to 37°C [32]. We used cultured differentiated podocytes, which express specific podocyte synaptopodin proteins, to investigate whether RANK was involved in podocyte injury with PA. Mouse podocytes were exposed to PA (25 µg/ml), which is known to induce podocyte injury in rodents. Confocal microscopy showed that the bulk of RANK localizes to the cell membrane and cytoplasm (Figure 4A). RANK immunofluorescence staining showed membrane localization in SJRH30 cells, which were RANK positive controls (Figure 4B). To determine whether RANK levels increased after PA (25 µg/ml) exposure, RANK mRNA was measured by real-time RT-PCR at 24 h and 48 h after PA exposure. A low abundance of RANK mRNA was detected in control cells that were not exposed to PA. By contrast, RANK was upregulated upon podocyte injury with PA, and the level of RANK peaked within 24 h. Densitometric analyses indicate a 1.8- and 1.5-fold induction of RANK at 24 and 48 h, respectively (Figure 4C). Whole-cell lysates were harvested 24 h or 48 h after PA injury, and RANK protein expression was determined by using Western blot analysis with RANK antibody. Densitometric analyses revealed that RANK was upregulated by 3.8- and 2.4-fold at 24 h and 48 h respectively after PA injury (Figure 4D). RANK immunoblotting revealed that RANK was upregulated in an autocrine manner upon PA injury in podocytes, reaching a maximum after 24 h PA treatment. RANK was confirmed by using Western blot analysis with RANK antibody in SJRH30 cells, which are RANK positive controls (Figure 4E).

### RANKL and RANK Protect Mouse Podocytes from Apoptosis

**Figure 5:**
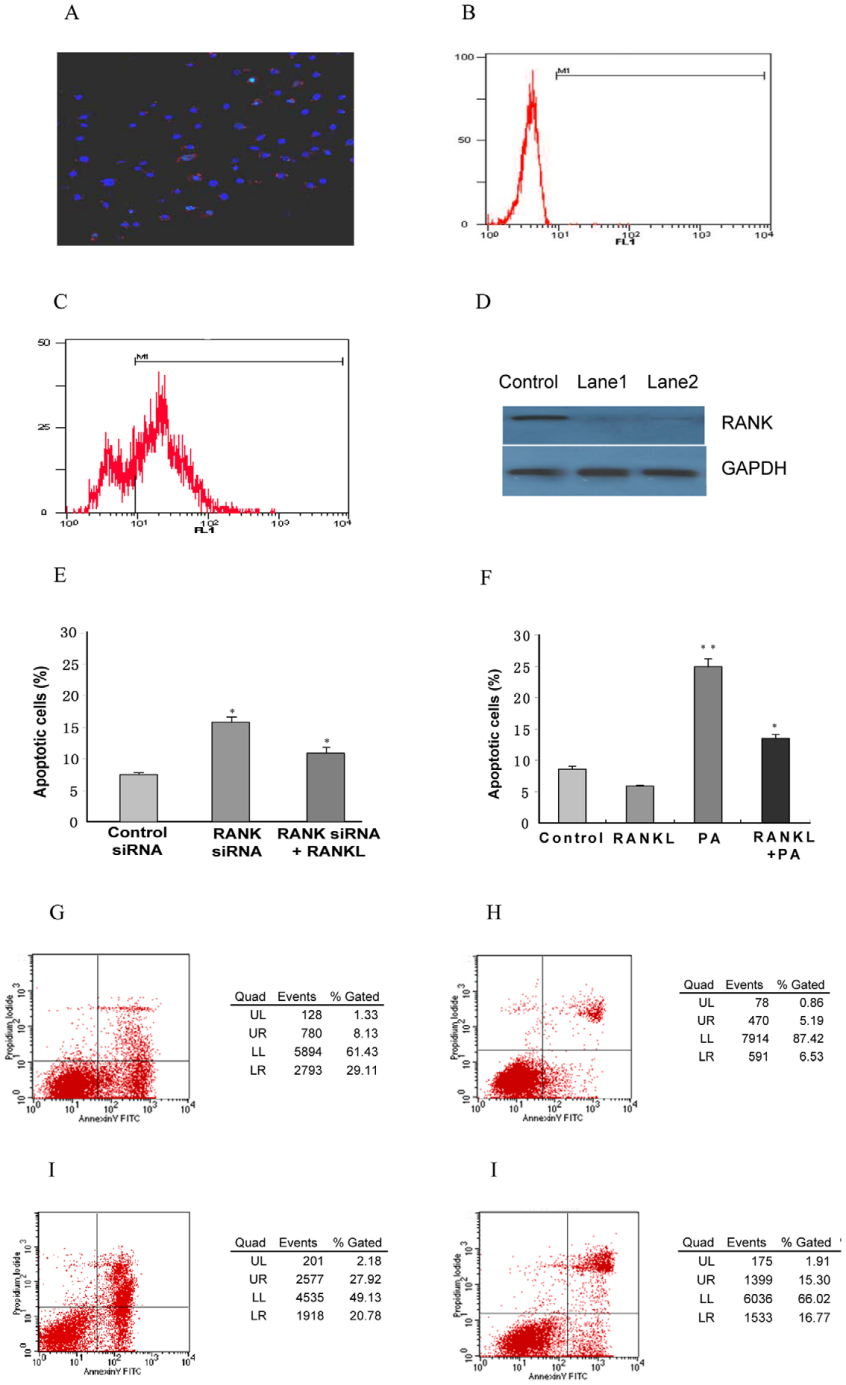
RANKL and RANK Protects Mouse Podocytes from Apoptosis. (A) Mouse podocytes were transfected with RANK siRNA at a concentration of 100 nM. (B) Flow cytometry fluorescence of podocytes not transfected with siRNA-Cy3 was measured as negative control; the value in podocytes was 4.1%. (C) Transfection efficiency was measured by counting fluorescence-positive cells by flow cytometry; the value in podocytes was 71.6%. (D) RANK immunoblotting of podocytes revealed that RANK was downregulated with RANK siRNA. RANK protein was low abundance in the podocytes 3 d after transfection (lane 1 and lane 2). (E) The percentage of apoptotic cells was measured by flow cytometry. In cells transfected with RANK siRNA, RANK knockdown was associated with mild increase in apoptosis compared with control siRNA (24.0±1.8% versus 22.8±1.1%; p>0.05) after podocytes were exposed to PA. However, RANKL (40 ng/ml) decreased PA-induced apoptosis of podocytes with RANK siRNA (RANKL 16.5±1.5% versus control 22.8±1.1%). (F) Quantification of apoptosis of podocytes with PA and RANKL. Apoptosis was measured by flow cytometry in control podocytes without PA induction (G) (8.7±0.97%) and podocytes that were exposed to RANKL (40 ng/ml) without PA after 48 h (H) (5.7±0.81%). (I) PA increased the apoptosis of podocytes. Apoptosis was measured by flow cytometry of podocytes exposed to PA (25 µg/ml) for 48 h (26.3±3.6%). (J) Exogenous RANKL protected podocytes from PA induction apoptosis. RANKL (40 ng/ml) decreased PA-induced apoptosis (15.5±2.2%). All the experiments were conducted in three times. **Compared with control, p<0.01; *Compared with control, p<0.05.

To better understand RANK function in immortalized mouse podocytes, we used an *in vitro* RANK knockdown system to determine whether RANK was necessary for podocyte survival [33]. We consistently achieved close to 71.6% siRNA transfection efficiency in podocytes, as visualized by transfecting a fluorescently tagged Cy3-RANK siRNA. Flow cytometry fluorescence of podocytes not transfected with siRNA-Cy3 was measured as negative control; the value in podocytes was 4.1% (Figure 5A,5B and 5C). Knockdown of RANK was determined to be maximal between days 3 and 4 after transfection (Figure 5D). We did not observe any morphologic changes between cells with or without RANK siRNA knockdown during the 4 d after transfection. To test whether RANK was involved in the apoptosis of podocytes *in vitro*, we studied podocytes apoptosis before and after stable knockdown of RANK with siRNA. The knockdown of RANK alone did not induce podocytes apoptosis, but increased mildly the apoptosis of podocytes exposed to PA. However, RANKL reduced apoptosis of podocytes transfected with RANK siRNA exposed to PA compared with control siRNA (RANK siRNA 16.5±1.5% versus control siRNA 24.0±1.8%, p<0.01, Figure 5E).

To investigate whether RANKL was associated with podocytes apoptosis [34], we pretreated differentiated mouse podocytes with exogenous RANKL (40 ng/ml) before PA (25 µg/ml) treatment, and measured apoptosis. Quantification of apoptosis of podocytes with PA and RANKL is shown in Figure 5F. The percentage of apoptotic cells was measured by flow cytometry (FAC-Scan) in control podocytes (8.7±0.97%, Figure 5G) and podocytes exposed to RANKL (40 ng/ml) after 48 h (5.7±0.81%, Figure 5H). PA (25 µg/ml) exposure increased apoptosis of podocytes after 48 h (26.3±3.6%, Figure 5I). Exogenous RANKL protected podocytes from PA-induced apoptosis (15.5±2.2%, Figure 5J). All the experiments were conducted in three times.

## Discussion

PAN is a common model for the study of the development and progression of glomerular injury [28]. PA induces flattening of the podocyets, loss of foot processes, and a focal loss of podocytes along with the GBM. These changes indicate that podocyte injury is the primary pathogenic event in puromycin aminonucleoside nephrosis.

Receptor activator of NF-kappaB and its ligand have been identified as important regulators of interactions between T cells and dendritic cells [19]. RANK and RANKL have emerged as important factors in epithelial cells growth and lymphoid tissue cells proliferation. RANK affects a great variety of epithelial cells of different organs. It is required for the formation of lactating mammary glands [35]–[36], epithelial cells of the hair follicle and the interfollicular epidermis, thymic medullary epithelial cells [37], and intestinal microfold cells [38] and it has been implicated in the growth and metastasis of prostate [39] and mammary epithelial cancers [40]. Podocyte is visceral glomerular epithelial cell, which is a terminally differentiated cell that lines the outer aspect of the GBM. We have studied whether RANK plays a functionally important role in the visceral glomerular epithelial cell. The present study demonstrated that RANK was upregulated in the glomerular podocytes of rat kidney after PA injection. RANK co-localized with synaptopodin which is a marker protein of podocyte. We defined that the subcellular localization of RANK was on the top of the foot process membrane and the cytoplasm of podocytes with immunogold analysis. We also found that the RANKL in the kidney of rats with PAN had significantly increased compared with the control. Podocytes are injured in many forms of human glomerular disease, including minimal change disease, FSGS, membranous glomerulopathy, diabetes mellitus, IgA nephropathy, and lupus nephritis [11].The present study demonstrated that RANK and RANKL were upregulated in human glomerular disease (FSGS, IgA nephropathy and MN) compared with the control. However, there was no expression of RANK in the sclerosis of FSGS. These findings suggest RANKL/RANK interaction plays a pivotal role in the pathogenesis of podocyte injury.

The tumor necrosis factor (TNF) family member RANKL and its receptor RANK are important regulators of T cells [19]. LPS-induced foot process effacement and proteinuria is a typical model of podocyte acute injury in mice [9]. To determine whether the LPS-induced RANK derives from podocytes or the activation of T cells, we checked RANK in the LPS-treated SCID mice, which are devoid of T and B cells [30]. The experiments demonstrated that the induction of RANK in podocytes was not mediated through the action of T or B cells. The results suggest a link between RANK overexpression and podocyte injury that is independent of lymphocyte infiltration or activation.

Despite the importance of podocytes in renal physiology and as a target of glomerular disease [5], the underlying molecular mechanisms that control the survival or apoptosis of podocytes have not been fully elucidated. The present study demonstrated that RANK was upregulated in an autocrine manner with PA injury, reaching to a maximum after 24 h PA treatment. We demonstrated that PA enhanced podocytes apoptosis and the effect was dose and time dependent. PA also induced podocytes necrosis at higher concentrations (data no shown). Application of exogenous RANKL significantly inhibited podocytes apoptosis induced by PA. Likewise, the selective silencing of RANK in podocytes exacerbated the proapoptotic effects of PA. Several studies have demonstrated that RANK and RANKL were involved in cell apoptosis. Hong-Hee Kim and colleagues found that RANK was expressed by endothelial cells, and RANKL suppressed apoptosis of primary cultured endothelial cells through the PI 3′-kinase/Akt signal transduction pathway [24]. Schramek et al. reported that RANKL, through interaction with RANK on mammary epithelial cells, drove these cells into the cell cycle and protected mouse and human mammary gland epithelial cells from apoptosis [41].

Podocytes respond to injury by enacting coordinated genetic programs. These responses can modify the cell in an adaptive manner against the noxious stimuli and to promote their recovery from an injury [11]. In response to injury, podocytes secrete PTHrP [42], heme oxygenase-1 [14], CD74 [43], COX-2 [44]–[45], B7-1 [9], and glial cell line-derived neurotrophic factor [16], which is a survival growth factor of injured podocytes. RANKL is found to be expressed in embryonic kidney tissue, but it is absent in kidney of adult [28]. We found that RANKL was overexpressed in response to podocyte injury. The study suggests that when podocytes are damaged, embryonic protein expression will recurrent. Given that both RANKL and RANK are upregulated in injured podocytes, RANKL tends to act in an autocrine manner to support podocyte survival.

Although the expression of RANK and RANKL is induced in podocytes with PA, the underlying molecular mechanisms and signaling pathways of RANKL-RANK complex are incompletely understood. We demonstrated that RANK-RANKL complex was important for response to podocyte injury *in vitro*. However, future studies will be needed to define the RANK-RANKL complex physiological and pathophysiological functions in kidney disease with gene knockout mice model *in vivo*.

In summary, the upregulation of RANKL and RANK, in combination with the significant protective effects of RANKL, indicates that RANK is part of an adaptive, recovery response to podocyte injury. This is the first observation that RANKL, acting through RANK, functions in an injury paradigm in the kidney. These data raise the exciting therapeutic possibility of giving exogenous RANKL to patients with glomerular disease that is characterized by a loss of podocytes, such as membranous nephropathy and focal segmental glomerulosclerosis.

## Methods

### Animals

Male Sprague-Dawley rats that weighed 110∼130 g were fed a standard diet. Rats were divided into the control and PAN groups. PAN was induced by a single intravenous injection of PA (Alexis) at 10 mg/100 g body weight, diluted in 0.9% saline [46]. Control animals received saline only. Urinary protein was measured with nephelometry (Beckman Instruments). On days 3, 7, and 14 after PA injection, rats (n = 6 in each group at each time point) were killed under anesthesia, and kidneys were harvested and frozen in liquid nitrogen or fixed in a 4% paraformaldehyde solution.

SCID mice were obtained from the Jackson Laboratory. SCID mice urinary protein was analyzed by a urine dipstick before the animals were injected intraperitoneally with either 200 µg LPS [9] in a total volume of 200 µl, or equal volumes of sterile LPS-free PBS (PBS, n = 3; SCID, n = 3). In SCID mice (n = 3), urine was collected before and 24 h after LPS injection and analyzed by the dipstick. All measurements were performed in duplicate.

This study was carried out in strict accordance with the recommendations of the Guide for the Care and Use of Laboratory Animals of Sun Yat-Sen University. All animal procedures were approved by the Animal Experiment Committee of Sun Yat-Sen University (protocol number 10100341).

### Patients

Diseased kidney tissues were obtained from needle biopsies of 42 patients with proteinuria. The kidney tissues were obtained with informed consent to be used for research purposes after the diagnostic workup was completed. The study’s protocol was approved by Guangdong General Hospital Ethics Committee (protocol number YY01023). Of the 42 patients with proteinuria, the pathological diagnosis included IgA nephropathy (IgAN), membranous nephropathy (MN), and focal segmental glomerular sclerosis (FSGS). Normal kidney tissues were obtained from surgical nephrectomy necessitated by the presence of renal tumors (n = 3). Podocyte foot process effacement was seen in kidney tissues from all patients with proteinuria, as demonstrated by electron microscopy. No pathological findings were observed in normal kidney tissues.

### Cell Culture and Transient Transfection

An immortalized murine podocyte cell line [32] between passages 12 and 20 was provided by Dr. FR Danesh. At a permissive temperature of 33°C, the cells remain in an undifferentiated proliferative state, whereas raising the temperature to 37°C results in growth arrest and differentiation to the parental podocyte phenotype. Undifferentiated podocyte cultures were maintained at 33°C in RPMI 1640 medium (Life Technologies) with 10% fetal bovine serum (FBS; Life Technologies). Once cells had reached 70 to 80% confluence, they were cultured at 37°C for at least 7 d before use, by which time full differentiation had taken place. For experiments, cells were cultured in serum-free medium 24 h before the addition of RANKL (40 ng/ml) (R&D Systems) and PA (25 µg/ml) (Alexis) throughout the experiment. Podocytes were exposed to different concentrations of RANKL and PA for 24 or 48 h. Podocytes were collected and gently resuspended in 500 µL binding buffer. Annexin V-FITC (BioVision) and PI (BioVision) were added and incubated with cells in the dark for 10 minutes. At the end of the incubation, the cells were analyzed by flow cytometry (FAC-Scan; Becton Dickinson) to discriminate between live and apoptotic cells.

SJRH30 cells are human rhabdomyosarcoma cell lines, which are RANK positive controls (American Type Culture Collection, ATCC). SJRH30 cells were cultured at 37°C and 5% CO_2_ in 25 ml flasks in a culture medium composed of RPMI-1640 medium (Life Technologies) supplemented with 10% fetal bovine serum (FBS; Life Technologies).

RANK small interference RNA (siRNA) knockdown was performed by using transient transfection of pooled, functionally validated Cy3–labeled RANK siRNA (Invitrogen) [16]. Podocytes that were differentiated for 10 to 12 d were maintained at 10% FBS/RPMI as described above, and transfected using the RANK siRNA transfection reagent (Neuromics). For determination of the transfection efficiency, a Cy3–labeled RANK siRNA was analyzed by flow cytometry. Western blot analysis for RANK was performed with samples from cells 24 to 96 h after the transfection. Several concentrations of RANK siRNA (20, 40, and 60 nM) were tested to determine optimal knockdown conditions.

### Immunohistochemistry and Immunofluorescence

Human and murine renal tissues were cut into 5 µm cryosections that were fixed in acetone for 10 min at −20°C, washed with phosphate-buffered saline (PBS), then treated with 0.5% Triton X-100 for 15 min, and blocked with 5% BSA for 30 min. Podocytes on coverslips were washed with PBS, then fixed with 4°C acetone and blocked with 5% BSA. For indirect immunofluorescence staining of kidney sections, cryosections were stained with antibodies against Synaptopin (1∶50, Santa Cruz Biotechnology) overnight at 4°C. After washing, the sections were incubated in FITC-conjugated anti-IgG secondary antibody (1∶100, Santa Cruz Biotechnology) for 60 min. The primary antibodies that were used for immunofluorescence studies also included anti-RANK antibody (RANK H-300, 1∶50, Santa Cruz Biotechnology) and anti-RANKL antibody (1∶50, abcam). RANK (H-300) is recommended for detection of RANK of mouse, rat and human origin by Western Blotting (dilution 1∶1000), immunofluorescence (dilution 1∶50). Antigen–antibody complexes were visualized with secondary antibodies (1∶100 or 1∶200, Santa Cruz Biotechnology) conjugated with Rhodamine. We added second antibody dilution times (1∶200), and reduced nonspecific staining. We used positive control SJRH30 cells, which were expressed RANK on the plasma membrane. Sections were viewed and imaged with confocal microscopy (Zeiss LSM510).

### Transmission Electron Microscopy (TEM)

For transmission electron microscopy, ultrathin sections (60 to 100 nm) were cut from cortical kidney tissue samples embedded in epon resin, using an ultramicrotome (Leica), collected on copper grids, and stained with uranyl acetate and lead citrate. Ultrathin sections were stained with uranyl acetate for 10 min and subsequently in Reynolds lead citrate for 2 min. Ultrastructural analysis was performed by transmission electron microscopy [47].

### Real-Time Polymerase Chain Reaction

Total RNA was extracted from human renal biopsy specimens using the Qiagen RNeasy Micro kit (Qiagen) according to the manufacturer’s protocol. Total RNA was extracted from rodent renal cortex tissues using a TRIzol kit (Invitrogen) according to the manufacturer’s instructions. First-strand cDNAs were synthesized using oligo(dT)_15_ with Superscript reverse transcriptase (Invitrogen). The housekeeping gene GAPDH was used as an internal control. To quantify the transcript copy of target genes and GAPDH in each sample, DNA plasmid standards corresponding to target genes and GAPDH were prepared and used in the real-time quantitative PCR assay. Real-time PCR was performed using the MJ Opticon II Quantitative PCR System (MJ Research, Waltham, MA, USA) with the following thermal cycling profile: 95°C for 5 min, followed by 40 cycles of amplification (95°C for 20 s, 60°C for 25 s, and 72°C for 20 s). The absorption values of the SYBR Green I fluorescence in each tube were detected at the end of each cycle. Quantification in each sample was done by expressing the ratio of transcript copy number of target genes (RANK and RANKL) versus that of GAPDH. PCR primers for the genes of interest used in this study are shown as follows: rat RANKL (Accession NM 057149) forward: GCT CAC CTC ACC ATC AAT GCT; reverse: GGT ACC AAG AGG ACA GAC TGA CTT TA; rat GAPDH (Accession M17701)forward: CTC CCA TTC CTC CAC CTT TG; reverse: CCA CCA CCC TGT TGC TGT AG; mouse RANK (Accession BC019185) forward: GCT GGC TAC CAC TGG AAC TC; reverse: TGT GCA CAC CGT ATC CTT GT; mouse GAPDH (Accession M32599) forward: CGT GTT CCT ACC CCC AAT GT; reverse: TGT CAT CAT ACT TGG CAG GTT TCT; human RANK (Accession M32599) forward: CCC GTT GCA GCT CAA CAA G; reverse: GCA TTT GTC CGT GGA GGA A; human RANKL (Accession NM 033012) forward: ACG CAG TGA AAA CAC AGT T; reverse: TGC CTC TGG CTG GAA ACC; human GAPDH (Accession M33197) forward: AAA TTC CAT GGC ACC GTC AA; reverse: AGG GAT CTC GCT CCT GGA A.

### Western Blot Analysis

Protein expression of RANK and RANKL were determined by Western blot analysis [48]. Briefly, renal tissue was homogenized in 1 ml of tissue lysis buffer. Samples were centrifuged at 3,000 g for 15 min, and the supernatants were assayed. After being mixed with SDS-PAGE (NuPAGE) sample buffer and boiled for 5 min, samples were electrophoresed on 10% SDS polyacrylamide gels and transferred to PVDF membranes (Millipore) for 2 h at 40 V. Membranes were blocked for 30 min with Tris-buffered saline that contained 5% BSA (5% BSA/TBS) and incubated with diluted primary antibody including anti-RANK (1∶1000, Santa Cruz Biotechnology), anti-GAPDH (1∶1000, Santa Cruz Biotechnology) and RANKL (1∶1000, abcam) overnight at room temperature in 5% BSA/TBS that contained 0.05% Tween 20. The membranes were washed and developed using the enhanced chemiluminescence system (Applygen Technology).

### Statistical Analysis

Results are expressed as means ± SE. The difference between groups at one time point was assessed by Student’s t-test. Comparisons were made by analysis of variance using the computer software PEMS 3.1 for Windows software (Package for Encyclopaedia of Medical Statistics, Chengdu, China). Differences were considered significant if the p value was less than 0.05.
